# Maternal health workers’ knowledge, practices, and influencing factors in screening and managing perinatal mental health conditions in primary healthcare facilities in Addis Ababa, Ethiopia: a qualitative study

**DOI:** 10.1186/s12913-026-14284-w

**Published:** 2026-03-05

**Authors:** Michael Bimrew, Sally Field, Lenka Beňová, Marie Meudec, Samson Gebremedhin, Abdulhalik Workicho, Delayehu Bekele, Andamlak Gizaw Alamdo, Anteneh Asefa

**Affiliations:** 1 Gulele Sub City Health Office, Addis Ababa City Administration, Addis Ababa, Ethiopia; 2 Perinatal Mental Health Project, Centre for Public Mental Health, Department of Psychiatry and Mental Health, University of Cape Town, Cape Town, South Africa; 3 Department of Public Health, Institute of Tropical Medicine, Antwerp, Belgium; 4 Department of Epidemiology and Population Health, London School of Hygiene and Tropical Medicine, London, UK; 5 School of Public Health, Addis Ababa University, Addis Ababa, Ethiopia; 6 Maternal, Child and Adolescent Health Service Lead Executive Office, Ministry of Health, Addis Ababa, Ethiopia; 7 Harvard University T. H. Chan School of Public Health Fenot Project , Addis Ababa, Ethiopia; 8 Department of Obstetrics and Gynecology, St Paul’s Hospital Millennium Medical College, Addis Ababa, Ethiopia; 9 Department of Health Service Management, Health Promotion, Reproductive Health and Nutrition, School of Public Health, St. Paul’s Hospital Millennium Medical College, Addis Ababa, Ethiopia

**Keywords:** Perinatal mental health, Postpartum depression, Health workers, Capacity, Integration

## Abstract

**Background:**

Poor perinatal mental health is a significant public health issue, especially in low- and middle-income countries. In sub-Saharan Africa, perinatal mental health conditions affect more than one in five women. Ethiopia’s mental health strategy mandates the integration of mental health services into primary care. However, screening and treatment for perinatal mental health conditions remain largely limited due to various challenges, including weak integration into maternal and child health services. This study aimed to explore maternal health workers’ knowledge, practices, and perceived factors influencing the screening and management of perinatal mental health conditions in primary healthcare facilities.

**Methods:**

This qualitative exploratory descriptive study was conducted between August 2023 and March 2024 in ten primary healthcare facilities and the responsible sub-city health department in Addis Ababa. We conducted 25 in-depth interviews with frontline maternal health workers, facility managers, and program coordinators. Interviews were audio-recorded, transcribed verbatim, and translated from Amharic to English. Data were analyzed using Braun and Clarke’s six-phase inductive thematic analysis approach, supported by ATLAS.ti software. Two authors independently coded the data, and themes were developed and refined through team consensus. The Consolidated Criteria for Reporting Qualitative Research (COREQ) checklist was used to guide reporting.

**Results:**

We identified four interconnected themes. 1) **Foundational knowledge**,** misconceptions**,** and attitudes towards perinatal mental health**: maternal health workers demonstrated foundational knowledge of perinatal mental health conditions, but this was inconsistent. Notable misconceptions included the conflation of postpartum depression with the ‘baby blues’ and underestimating its local prevalence. Despite these gaps, participants held strongly favorable attitudes towards the integration of perinatal mental health services; 2) **Current** **practices in** **screening and management** **of perinatal mental health conditions:** practices were informal and reactive, as none of the ten facilities used validated screening tools or followed standardized protocols. Assessment relied on opportunistic observation and patient-reported complaints, while management was limited to basic counseling and unreliable referral pathways; 3) **Perceived readiness and capacity gaps**: there was a disconnect between managerial confidence and frontline workers’ self-reported unpreparedness. Managers assumed readiness, whereas frontline health workers reported significant capacity gaps in their ability to provide integrated perinatal mental health services; 4) **Systemic and socio-cultural barriers to implementation**: these included overwhelming workloads, inadequate infrastructure, a shortage of mental health professionals, pervasive stigma, and the spiritual attributions of mental illness, which collectively constrained effective care.

**Conclusions:**

This study highlights critical gaps in maternal health workers’ capacity to provide perinatal mental health services, emphasizing the need for targeted training, standardized tools, and systemic support to effectively integrate perinatal mental healthcare into the routine maternal and child health services.

**Supplementary Information:**

The online version contains supplementary material available at 10.1186/s12913-026-14284-w.

## Introduction

The global burden of mental health conditions is high, leading to significant negative impacts at individual, family, and societal levels. According to the Institute for Health Metrics and Evaluation, 13.9% of the world’s population experienced a mental health condition in 2021 [1]. This burden is often exacerbated in low- and middle-income countries (LMICs) due to conflict, poverty, and limited access to services [2, 3].

A critical gap in care exists for perinatal mental health (PMH) conditions, which include depression and anxiety occurring during pregnancy or the postpartum period. When left unaddressed, these conditions are associated with increased morbidity and mortality for both mothers and their children [3, 4]. The burden of perinatal depression and anxiety is disproportionately high in LMICs. A systematic review of 589 studies conducted across 51 LMICs, 48 of which were in sub-Saharan Africa (SSA), reported a pooled prevalence of perinatal depression of 25.5% [5]. In Ethiopia, the focus of this study, the pooled prevalence is similarly high, ranging from 21% to 26% [6–8]. Despite this high burden and the negative consequences of poor PMH, which include impaired maternal-infant bonding and increased risk of infant developmental delays [9], PMH care remains largely neglected [10, 11].

Perinatal depression and anxiety arise from a complex interplay of risk factors operating at individual, community, and structural levels. At the individual level, risk factors include unwanted pregnancy, history of depression, and lack of social support [12]. Community-level barriers, such as stigma and the normalization of maternal distress, can discourage women from seeking care [12]. At structural level, fragmented healthcare systems often fail to integrate culturally sensitive routine screening for PMH conditions into standard antenatal and postnatal care [13]. These gaps are further exacerbated by policy shortcomings, workforce shortages, and financing challenges, that lead to an over-reliance on undertrained community health workers for perinatal care [14].

An effective response requires the integration of PMH care into existing health services [15]. The World Health Organization recommends integrating PMH services into maternal and child health (MCH) services to leverage routine antenatal and postnatal contacts for early detection and management of PMH conditions [16]. Ethiopia’s national mental health strategy aligns with this approach by adopting a primary health care-based approach that mandates the integration of mental health services into routine care to promote equitable and accessible coverage. This strategy emphasizes task-sharing and the adoption of the WHO’s Mental Health Gap Action Programme (mhGAP) to train non-specialist health workersin integrated mental health services delivery [17, 18].

Ethiopia’s health care system is organized into three tiers: primary, secondary, and tertiary. In the context of MCH service provision in Addis Ababa, the primary tier consists of health centers with an inpatient capacity of up to 12 patients, private clinics, and MCH centers. These provide basic health services including antenatal care, childbirth, postnatal care, and child immunization. Although not universally available, some health centers have dedicated mental health outpatient departments staffed by mental health professionals. The secondary tier comprises general hospitals that provide advanced MCH care, while the tertiary tier consists of comprehensive specialised hospitals that provide care for complicated conditions, including mental health conditions requiring specialist care [19].

However, there is limited evidence from Ethiopia on the capacity of maternal health workers to provide integrated PMH services, or on the challenges they face in screening for and managing PMH conditions within the primary healthcare structure. The aim of this study was to explore maternal health workers’ knowledge, practices, and perceived factors influencing the screening and management of PMH conditions in primary healthcare facilities in Addis Ababa, Ethiopia.

## Methods

### Study design

We conducted a qualitative exploratory descriptive study between August 2023 and March 2024. We used the Consolidated Criteria for Reporting Qualitative Research (COREQ) checklist in the reporting of this study [20].

### Study setting

This study was conducted in ten selected primary health care facilities: eight public health centers, one private not-for-profit health center, and one private MCH specialty clinic in Gulele sub-city, Addis Ababa, Ethiopia. In 2023, there were 98 health centers, 13 public hospitals, and two military force hospitals across the 11 sub-cities of Addis Ababa. During the study period, there were 44 health facilities in Gulele sub-city: two tertiary hospitals, 10 public health centers, one private not-for-profit health center, and 31 private clinics. In Addis Ababa, Health centers are also mandated to coordinate the urban health extension program, in which urban health extension workers play a vital role in providing community-based health services, including preventive MCH services [19].

### Characteristics of participants

Twenty-five key informants participated: 23 from ten facilities and two from the Sub-City Health Office. Seventeen of the key informants were frontline maternal health workers from the antenatal, labor/childbirth, and postnatal care units, and six were facility-based team leaders or heads. Two maternal health program coordinators from the Gulele Sub-City Health Office were also purposively included to provide oversight and program implementation perspectives at the sub-city level. These officers oversee all facilities in the sub-city, including but not limited to the ten selected for this study (Table 1).

### Sampling methods

We used a two-tiered, purposive sampling strategy with maximum variation to capture diverse perspectives. Firstly, at the facility level, we ensured diversity by purposively selecting primary health facilities from both the public and private sectors within Gulele Sub-city. Secondly, within these chosen facilities, we recruited individual participants to maximize variation across three key dimensions: 1) professional role and hierarchy (from frontline maternal health workers and team leaders to facility heads and sub-city program coordinators), 2) professional discipline, and 3) years of experience. This approach was complemented by a sequential, bottom-up order of data collection, with frontline staff being interviewed first to ground our understanding, before team leaders, facility heads, and program coordinators were engaged, thereby ensuring that our findings reflected the full range of contexts and viewpoints within the maternal health system.

Recruitment continued until the research team agreed that thematic saturation had been reached across the key participant categories, the point at which new data yielded no further thematic insights. Key-informant interviews with 23 participants from health facilities was sufficient to achieve thematic saturation, given the research aim and the depth of data obtained through semi-structured interviews. We subsequently conducted two additional key-informants with maternal health program coordinators from the Gulele Sub-City Health Office to incorporate a health system management perspective, as these officers oversee all facilities in the sub-city, including those selected for this study.

The inclusion criteria encompassed health workers who were currently employed in the maternal health units of the selected health facilities, as well as maternal health team leaders and facility heads (e.g. Medical Directors and Chief Executive Officers) from these facilities. Key maternal health officers from the Gulele Sub-city Health Office were also included, specifically the MCH Officer and the sub-city MCH team leader. Individuals were excluded from the study if they were not directly involved in maternal health service provision within the selected health facilities, were absent during the data collection period, or declined to provide informed consent.

### Data collection methods

The first author (MB) conducted face-to-face interviews in private rooms at participants’ workplaces to ensure both convenience and confidentiality. This familiar setting facilitated open discussion; however, the authors remained reflexive to the possibility that conducting interviews on-site could influence participants’ responses despite assurances of confidentiality and voluntary participation. Interviews lasted on average 30–40 minutes and were audio-recorded with participants’ consent.

### Data collection tools

Data were collected using a semi-structured interview guide developed by the research team in a multiphase process (Supplementary file 1). First, a critical review of the literature on the integration of PMH into MCH services in low-resource settings was conducted to identify key thematic areas of exploration. Secondly, we reviewed national policy and strategic documents on mental health, and maternal, sexual, and reproductive health to refine the thematic areas in light of identifying potential barriers and facilitators to the WHO’s stepped care approach to integrating PMH into MCH services [16]. Finally, the question guides were revised based on the preliminary findings of the MISPOD study: ‘Is MIStreatment of women during facility-based childbirth an independent risk factor for POstpartum Depression in Ethiopia and Guinea?’ [21]. The study involved a longitudinal survey of 373 women from pregnancy to up to 16 weeks postpartum in Addis Ababa to assess the independent association between the mistreatment of women during childbirth in health facilities and postpartum depression. The guide was originally prepared in English and later translated into Amharic; the Amharic version was used for data collection.

### Data analysis

Audio recordings were transcribed verbatim and translated into English by MB and then checked for accuracy by AA. Nine of the 25 interviews were returned to participants for member-checking to confirm that their views and experiences were accurately reflected. MB, SF and AA analyzed data using Braun and Clarke’s inductive thematic analysis approach [22], supported by ATLAS.ti software. The analysis followed six iterative phases: (1) familiarization with the data through repeated reading of transcripts; (2) systematic generation of initial codes by two authors (MB and SF) to identify meaningful data segments; (3) searching for themes by collating related codes; (4) reviewing and refining initial themes; (5) defining and naming themes; and (6) producing the narrative report. Data were organized into three hierarchical levels: overarching themes, sub-themes, and where relevant sub-sub-themes, to capture the complexity and nuance of paticipants’ accounts. Themes and sub-themes were iteratively reviewed and refined by MB, SF, and AA to ensure coherence and accuracy. Throughout the analysis, particular attention was given to divergent views and perspectives, which were examined and discussed until consensus was reached. In the final stage, the researchers interpreted the themes in relation to the research questions, drawing on existing literature on PMH service integration.

### Reflexivity of the first author

As the first author of this study, my (MB) positionality is shaped by my professional role and personal experience of working within the MCH system in Gulele sub-city, Addis Ababa, Ethiopia, the setting of this study. As a member of the MCH team in the sub-city health department, I provide technical support to the health facilities included in this study. This close engagement provides me with an insider’s view of the practical realities of MCH services delivery in this setting. Through my work, I have frequently witnessed how PMH is being overlooked, which led me to propose this study. On the other hand, my position as a technical support provider within the sub-city health system could introduce potential bias, as participants might perceive me as someone appraising their performance and respond in a way that they believe aligns with ideal expectations. To minimize this potential bias, I took several steps to ensure the integrity and objectivity of the study. Firstly, I assured participants that their responses would be kept confidential and would not affect their professional standing or relationships within the health system. Secondly, I emphasized that the purpose of the study was to genuinely understand their experiences and challenges, without judgment or repercussions. Thirdly, I took a neutral and non-directive approach during the interviews to enable participants to express their views freely. Finally, I engaged in reflexive practices throughout the research process, continually reflecting on my role and its potential impact on the study to ensure that my positionality did not unduly influence the findings.

## Results

### Description of participant characteristics

Four-fifths (*n* = 20) of the participants were midwives by profession, and the mean service year of professional experience was 8.4 years (Table 1).

**Table 1:** Participants’ profiles

Health facility (HF)	Participant (*P*)	Qualification	Position	Service year
HF1	P1	BSc midwife (BSC-MW)	Frontline health worker: ANC, PMTCT, childbirth and PNC	3 years
	P2	BSc midwife	Frontline health worker: ANC, PMTCT, childbirth and PNC	7 years
	P3	MSc Health Services Management (MSc-HSM)	Medical director	9 years
HF2	P1	Diploma in midwifery (Dip-MW)	Frontline health worker: ANC, PMTCT, childbirth and PNC	9 years
	P2	BSc midwife	Frontline health worker: ANC, PMTCT, childbirth and PNC	10 years
	P3	BSc midwife	Frontline health worker: childbirth and PNC	9 years
HF3	P1	BSc midwife	Frontline health worker: ANC and PMTCT	8 Years
	P2	BSc midwife	Frontline health worker: childbirth and PNC	8 Years
HF4	P1	BSc midwife	Frontline health worker: ANC, PMTCT, childbirth and PNC	4 years
	P2	BSc midwife	Frontline health worker: childbirth and PNC	3 years
	P3	BSc midwife	Frontline health worker: childbirth and PNC	2 years
HF5	P1	BSc midwife	Frontline health worker: childbirth and PNC	7 years
	P2	BSc midwife	MCH team leader	13 years
	P3	MSc Emergency surgery	Chief executive officer	10 years
HF6	P1	BSc midwife	Frontline health worker: childbirth and PNC	5 years
	P2	BSc midwife	Frontline health worker: childbirth and PNC	2 years
HF7	P1	BSc midwife	Frontline health worker: childbirth and PNC	10 years
	P2	BSc midwife	Frontline health worker: childbirth and PNC	6 years
HF8	P1	BSc midwife	Medical director	15 years
	P2	BSc midwife	Frontline health worker: childbirth and PNC	8 years
	P3	BSc midwife	Frontline health worker: childbirth and PNC	2 years
HF9	P1	Obstetrician and Gynecologist (OB-GYN)	Chief executive officer	18 years
HF10	P1	Diploma in midwifery (Dip-MW)	MCH and family planning team leader	8 years
Sub-city Health Office (SHO)	P1	Master of Public Health (MPH)	Maternal health and cervical cancer screening coordinator	15 years
	P2	Master of Public Health (MPH)	MCH team leader	20 years

*ANC: Antenatal care; MCH: maternal and child health; PMTCT: Prevention of mother-to-child transmission of HIV; PNC: Postnatal care

### Study findings

Our findings revealed significant challenges that health workers face in providing PMH care. These challenges are categorized into four key themes: (1) Foundational knowledge, misconceptions, and attitudes towards PMH; (2) Current practices in screening and management of PMH conditions; (3) Perceived readiness and capacity gaps; and (4) Systemic and socio-cultural barriers to implementation. These themes often overlapped, reflecting the complex and interconnected nature of the challenges and opportunities in providing PMH services.

## Theme 1: Foundational knowledge, misconceptions, and attitudes towards perinatal mental health

Participants described various PMH conditions they knew and have encountered in clinical practice. Their responses included descriptions of antepartum and postpartum depression and anxiety, with varying levels of detail about their symptoms and presentations.

### Knowledge of perinatal mental health conditions

Maternal health workers demonstrated a foundational knowledge of PMH conditions. Health workers described conditions such as antepartum and postpartum depression and anxiety, drawing on knowledge acquired through formal education, limited in-service training, self-directed learning, and, in some cases, personal experience. A participant explained, *“Let us start with antepartum depression*,* it manifests as an unexplained stress that persists or may extend into the postpartum period”* (BSC-MW HF4). Another participant described symptoms of antepartum depression as “*the lack of energy for daily activities*,* sleep disturbances*,* and loss of appetite”* (MSC-HSM HF1). While most participants emphasized emotional and functional symptoms, some associated perinatal depression with more serious manifestations. For example, a participant stated: *“Signs of perinatal depression may include reduced social interactions*,* hostility towards the newborn and oneself”* (BSC-MW HF2). 

Anxiety was another condition mentioned by participants, particularly in relation to first-time pregnancies, and was often described as co-occurring with depression. Participants often linked pregnancy-related anxiety to uncertainty and fear surrounding childbirth. A participant explained, *“Typically*,* they [women] may have mild conditions*,* such as anxiety*,* especially during their first pregnancy. I believe anxiety comes from not understanding the whole pregnancy process and the possible outcomes*,* and also the fear of labor pain”* (OB-GYN, HF9).

Participants’ foundational knowledge of PMH was primarily academic. As one participant noted: “*I had lectures during my undergraduate program*,* including course-work in psychiatry*” (BSC-MW HC2). Beyond pre-service training, knowledge was occasionally updated through limited in-service training opportunities: “*We had volunteer trainers from a mental health hospital… The training was provided to our staff by a psychiatrist and lasted for two days*” (BSC-MW HF5). Some participants also described broadening their understanding through personal initiative, including independent reading of scientific literature: “*my interest in mental health has led me to explore various research articles*” (BSC-MW HF8). For a few participants, personal experiences of motherhood provided a unique and experiential dimension to their understanding. One participant reflected, “*Having experienced postpartum depression myself*,* I can better relate to the struggles mothers face and recognize the signs more easily*.” (MPH SHO).

Participants futher identified a complex range of risk factors contributing to PMH conditions. Unplanned pregnancy was frequently cited as a key contributor: “*If a pregnancy is unplanned*,* there is a higher likelihood of encountering risks*,* where the partner may struggle to accept the pregnancy*,* and the woman may lack financial and mental preparedness*” (BSC-MW, HF4). Financial hardship was also consistently highlighted as a major stressor: “*The primary risk factor I would identify is economic deprivation. For example*,* a woman may be concerned about the cost of diapers*” (BSC-MW, HF1). A lack of social support, eapecially from partners and family members, was also perceived as critical as illustrated by one participant who stated, “*If a postpartum woman does not receive adequate attention and care from her family*,* especially her husband*,* she may become depressed*” (Dip-MW, HF10).

### Knowledge gaps and misconceptions about perinatal mental health conditions

A recurring sub-theme highlighted gaps, uncertainty, insufficient knowledge, and misconceptions in participants’ understanding of PMH conditions. Although many participants demonstrated basic awareness, several acknowledged limited formal training and uncertainty in identifying or differentiating PMH conditions. For example, one participant stated, *“We did not cover that [perinatal mental health] back in school*,* but I think postpartum depression is when a mother hates herself*,* when she cannot think clearly*,* when she is feeling down”* (*BSC-MW*,* HF2*). Some participants retrospectively interpreted past encounters as possible PMH condition. A participant stated, “*I never encountered these cases*,* but now thinking about it*,* I suspect one mother we assisted during childbirth. She was in dispute with her family and crying*,* so we advised her and her family so that she would not be depressed”* (*BSC-MW*,* HF4*).

Misconceptions regarding clinical distinctions were also reported, particularly confusion between postpartum depression with baby blues. A participant stated, *“Postpartum depression occurs three days after childbirth and baby blues after a week or five days”* (*BSC-MW HF1)*. Participants also perceived the burden of perinatal depression to be low, which may reflect under-recognition rather than true absence of cases. For instance, one participant said, “*Based on my experience of working in the postnatal care unit*,* I do not believe the magnitude of perinatal depression is high in this community*” (*BSC-MW*,* HF*7).

### Positive attitudes toward integration of perinatal mental health services

Despite existing knowledge gaps, participants overwhelmingly expressed that PMH should be part of routine maternal healthcare and emphasized the need for capacity building. The demand for formal training emerged as a dominant theme, with participants explicitly linking their perceived knowledge gaps to the absence of professional development opportunities. As one participant noted, *“We need training on Perinatal mental health issues because most of us lack the knowledge to assess these conditions”* (*BSC-MW HF7*). Others highlighted the importance of continuous on-the-job training and mentorship. For example, one participant suggested that, *“The sub-city health office should provide ongoing professional development for health workers”* (*BSC-MW HF1*).

Building on the expressed need for strengthening individual capacity, many participants emphasized that training alone would be insufficient without broader, system-level changes. As one participant explained, “*The Ministry of Health and the Regional Health Bureau should work on integrating mental health awareness and support into existing antenatal and postnatal care programs in order to ensure holistic maternal well-being*” (BSC-MW HF3).

## Theme 2: Current practices in screening and management of perinatal mental health conditions

This theme explored how PMH conditions are currently identified, assessed, and managed.

### Screening processes and the absence of standardized tools

Participants described the screening for PMH conditions as inconsistent and largely dependent on individual clinical judgment rather than standardized and evidence-based approaches. Across facilities, participants confirmed the absence of standardized screening tools or established protocols. As one participant stated, *“No*,* we do not have standardized tools. Our assessment is entirely based on the definitions and symptoms we have covered in midwifery school”* (*BSC-MW HF5*).

In the absence of standardized tools, health workers relied primarily on symptom recognition and non-specific history-taking. Assessment was typically initiated in response to patient-reported complaints or overt behavioral cues, rather than routine systematic inquiry. One participant explained, *“We do not have any tools. We just assess them based on their symptoms. We take detailed history from them… For instance*,* if a woman says she has trouble falling asleep or get headaches*,* we would suspect depression”* (*Dip-MW HF1*). Others described observing behavioral cues such as *“a mother isolating herself*,* avoiding breastfeeding*,* or refusing to talk”* (BSC-MW HF5).

This reliance on informal assessment resulted in a largely reactive approach, whereby mental health concerns were addressed only when symptoms became apparent or were explicitly raised by clients. As one participant acknowledged, *“To be honest*,* throughout my working experience… we never counseled pregnant or postpartum women about perinatal mental health conditions unless they complained of symptoms”* (Dip-MW, HF1). Similarly, another participant noted *“Mental health considerations mostly arise when a mother presents with symptoms. While we prioritize pregnancy danger signs*,* perinatal mental health is rarely assessed proactively”* (BSC-MW HF2).

### Limited management capacity and fragmented referral pathways

Participants described management of identified or suspected PMH conditions as limited and inconsistently implemented, largely shaped by facility resources, workload pressures, and the availability of mental health specialists.

For cases perceived as mild, the primary intervention involved basic psychoeducation and supportive counseling. However, participants noted that these interventions were often constrained by time limitations and competing clinical priorities. One participant explained, *“We offer counseling and reassurance for less severe cases*,* focusing on uplifting their spirits as part of the initial support”* (*Dip-MW HC10*). However, the scope was limited, with another participant admitting, *“Due to the high number of antenatal clients we serve*,* we do not advise mothers about depression unless they have complained of its symptoms”* (*BSC-MW HF1*). For cases perceived as severe, referral to a mental health trained health worker was described as the intended pathway. However, the effectiveness of this pathway was inconsistent and often hampered by a scarcity of mental health trained health workers and unclear referral chains. A participant from a facility with an available mental health professional noted, *“We now have a trained mental health professional at our facility that we can refer women to. If it is beyond our capacity*,* we will refer the woman to a psychiatrist for a better evaluation and management”* (*BSC-MW HF6*). In contrast, participants from facilities without dedicated mental health professional described relying on informal arrangements: *“For now*,* we do not have a specific referral system in place for this. However*,* the nearest hospital with a well-established psychiatric department is Saint Paul Hospital*,* so we usually refer patients there” *(*OB-GYN HF9*).

## Theme 3: Perceived readiness and capacity gaps

Participants expressed varying levels of readiness to manage PMH conditions, with frontline maternal health workers generally reporting lower confidence compared with facility managers. While some participants felt capable of providing initial support, many highlighted significant gaps in knowledge, training, and resources that limited their preparedness to effectively assess and manage PMH conditions.

### Disconnect in perception of preparedness

Managers in sub-city and health centers seemed confident that their staff had the knowledge and skills to recognize and respond to PMH conditions. As one manager stated, *“Our midwives… have learned about the problem*,* so if a woman shows signs of depression*,* I do not think they would overlook it”* (MPH SHO). Another manager noted, *“Although none of my team members have received specialized training… I believe they are vigilant and ready to screen for perinatal mental health issues”* (BSC-MW HF5).

In contrast, health workers frequently reported significant gaps in their readiness. One provider reflected, “*I do not think we are ready in terms of knowledge. For instance, you asked me earlier about the difference between baby blues and postpartum depression. I do not remember the difference. I answered you uncertainly because it has been a long time since I took the course at school*” (BSC-MW HF1). Another stated, “*In my opinion, there is no qualified health professional here to diagnose and treat postpartum depression*” (BSC-MW HF8).

### Conditional readiness and capacity building

Despite these challenges, some participants expressed sub-optimal readiness to manage PMH conditions, particularly in facilities with lower patient volumes or additional on-the-job training opportunities. One participant explained, *“In our facility*,* as we do not have a high flow of laboring mothers*,* we can allocate sufficient time for counseling and assessment*,* as they spend a considerable amount of time with us”* (Dip-MW HF9). Another participant rated their team’s ability to identify depressive symptoms saying, *“I would rate my team at about 75% out of 100% in their ability to identify women with depressive symptoms.”* (BSC-MW HF5).

In the absence of coordinated system-wide support, efforts to build capacity were were largely fragmented, and internally initiated. Some facilities organized their own training sessions to bridge perceived knowledge gaps. A manager described, “*In response*,* our management team organized discussions and invited a guest psychiatry professional to deliver a two-day training session for our health professionals*” (MSc Emergency surgery HF5). These well-intentioned efforts highlight a recognized need but also underscore the lack of a standardized approach to workforce development for PMH.

## Theme 4: Systemic and socio-cultural barriers to implementation

Participants described multiple systemic barriers that constrained the translation of knowledge and positive attitudes into effective PMH care. These barriers operated at the health system level through structural and organizational limitations, and within the broader socio-cultural context, where stigma and prevailing belief systems shape help-seeking behaviors and service utilization.

### Health system and organizational constraints

Multiple health system and organizational constraints were identified, including heavy workloads, limited infrastructure, and shortage of mental health professionals, all of which collectively constrained effective care.

#### Time constraints, workload and staff turnover

Participants reported that time constraints due to high patient loads limited opportunities for a thorough PMH assessment. One participant noted, *“Due to the uneven ratio of clients to health workers*,* we often lack the time to thoroughly assess every aspect of care”* (BSC-MW HF6). These challenges are compounded by institutional prioritization of physical and obstetric care over mental health. As another participant stated, “*Mainly*,* we are focused on the obstetric aspects*,* and mental health assessments are not considered part of the service because we have not received any guidance from higher authorities*.”(BSC-MW HF7).

Staff retention and motivation were also identified as challenges by the participants. High workloads and limited incentives contributed to burnout and turnover among healthcare providers. One participant explained, *“Skilled professionals leave for high-paying jobs. The benefits provided in government organizations may not meet their needs”* (MSc Emergency Surgery HF5). Another participant emphasized the need for motivation, stating: *“Motivation is critical*,* healthcare staff need encouragement to sustain this work”* (BSC-MW HF2).

#### Infrastructure and resource limitations

Participants reflected on how infrastructure and resource constraints hinder the provision of PMH services. They reported issues such as overcrowding. One participant stated that their health center lacks sufficient rooms to provide services properly: *“The primary challenge at our health center is the critical shortage of rooms to provide services”* (MSc-HSM HF1). Another participant explained how the lack of privacy compromises patient openness, stating, *“The antenatal room serves more than one client at a time. So*,* women may not feel free to tell us about their mental health issues”* (BSC-MW HF1).

#### Lack of mental health specialists and referral resources

The scarcity of mental health professionals and referral resources was another major challenge. Many participants reported that their facilities lacked psychiatric professionals, which made it difficult to provide specialized care. A participant shared, *“A notable challenge is the lack of psychiatric professionals in our facility. If we had a psychiatric professional onsite*,* they could provide training to other healthcare professionals”* (MSc Emergency surgery HF5).

### Socio-cultural challenges to the provision of perinatal mental health services

Beyond health system limitations, participants emphasized socio-cultural factors that shaped women’s willingness to disclose symptoms and seek care, thereby affecting the feasibility of PMH service integration.

#### Cultural beliefs and community stigma

Participants reported that cultural beliefs and community misconceptions surrounding mental health pose significant challenges. Many participants highlighted how stigma and traditional practices prevent women from seeking care. A participant explained, *“In our community*,* mental health conditions are often attributed to evil spirits or the evil eye*,* leading people to seek religious remedies such as holy water and rituals instead of medical interventions”* (BSC-MW HF5).

#### Limited community awareness

Low community awareness of PMH conditions further compounded these challenges. Participants emphasized the need for greater awareness creation activities and outreach to change perceptions and encourage help-seeking behaviors. One participant stated, *“There is a huge gap in awareness of maternal mental health issues in the community*,* and the role of health professionals in creating awareness is quite low”* (BSC-MW HF6). Another participant added, *“The main challenge within the community stems from the perception of mental health conditions. People suffering from mental illness may fear being stigmatized and labeled as ‘crazy*,*’ which makes it difficult for them to seek help”* (MPH SHO). Some participants proposed community-engaged approaches, suggesting collaboration with trusted local actors. One participant stated, “*When traditional healers understand mental health*,* they become allies rather than barriers to care*” (OB-GYN HF9).

## Discussion

The findings of this study reveal a fragmented and under-resourced system of PMH care within primary health facilities, where health workers face significant challenges in supporting women’s mental wellbeing during pregnancy and the postpartum period. Although maternal health workers acknowledge the importance of PMH, their capacity to provide integrated and high-quality care was constrained by intertwined systemic, individual, and socio-cultural barriers. Our analysis suggests that addressing PMH requires more than isolated training initiatives; rather, it calls for a system-level re-conceptualization of maternal healthcare in which mental wellbeing is embedded as a core component, supported by clear protocols, adequate resources, and sustained community engagement.

Health workers’ knowledge of PMH conditions was generally foundational but inconsistent, with many participants reporting difficulty distinguishing between transient conditions such as “baby blues” and clinically significant postpartum depression. These conclusions are based on participants’ self-reported descriptions and admissions of uncertainty during interviews, which revealed gaps in diagnostic acuracy. This knowledge gap likely contributes to the underestimation of PMH burden and missed opportunities for early intervention. Similar gaps in PMH knowledge and practice have been noted among health administrators in Ethiopia, suggesting that challenges extend beyond frontline health workers to managerial levels [23]. A recent study specifically identified the “*absence of providers trained on perinatal mental health*” and the “*absence of implementation tools*” as fundamental impediments to integration, directly corroborating with the findings of our study [24]. Comparable experiences in South Africa and other low-resource settings attribute these gaps to insufficient pre-service and in-service training on PMH [25]. Overall, this underscores systemic shortcomings in pre-service and in-service training frameworks, which currently do not provide the competencies needed to support integrated PMH care or workforce capacity development.

A critical finding was the absence of standardized care protocols and validated screening tools for PMH in health facilities, which led to inconsistent and reactive practices reliant on non-specific cues. This reliance on opportunistic detection rather than systematic screening is a well-documented driver of underdiagnoses in low-resource settings [26, 27] and reflects a broader systemic failure to operationalize policy commitments to mental health integration. This reflects a health system where PMH is not mandated, measured, or resourced, leaving well-intentioned health workers to improvise without support. The resulting uneven referral pathways, particularly challenging for private facilities, further illustrate how system fragmentation directly compromises care continuity [24].

The difference in perception between managers and frontline health workers regarding preparedness to screen for and manage PMH conditions highlights an improtant consideration for designing interventions for PMH integration. Failure to account for this results in the needs of health workers for capacity building being overlooked, as managers with overly positive assumptions about their team’s capacity may see little need to invest in further training [28]. In this regard, fostering consensus on implementation gaps, feasibility, resource availability, and role clarity is essential to support the successful integration of PMH into MCH services within primary care settings [29].

The cultural stigma and spiritual explanations for mental conditions identified in our study were not merely community-level obstacles but active determinants of care provision. This finding aligns with existing evidence showing that stigma, characterized by negative stereotypes, prejudice, and discrimination, significantly impedes to mental health care, deterring timely treatment and hindering optimal outcomes [30]. Stigma manifests differently across cultures, shaped by distinct societal norms and beliefs, and it has profound effects at both the individual and societal levels [30]. This highlights that PMH integration initiatives relying solely on clinical, facility-based approaches will be insufficient unless they also address key barriers throughout the entire care pathway, including those at the family and community levels. Effective PMH integration therefore requires culturally sensitive strategies that promote women’s and communities’ trust in the health system, while also recognizing the professional culture, well-being, and rights of health workers [11].

An important contextual factor relevant to the interpretation of our findings is the urban setting of Addis Ababa. Key challenges of urbanization such as overcrowding, pollution, violence, and reduced social support are associated with higher rates of anxiety and depression, particularly among women living in disadvantaged neighborhoods [31]. The shift toward nuclear family structuremay further reduce traditional postpartum support, compounding the financial strain and social isolation identified by participants as major risk factors for perinatal depression [31]. These urban-specific dynamics suggest that PMH integration efforts should be responsive to challenges related to poverty, housing insecurity, and declining informal support systems.

Despite the challenges identified, our study also identified promising opportunities for improving PMH care. Maternal health workers who had opportunities for in-service training demonstrated better confidence and interest in providing integrated PMH practices. Therefore, it is critical to build the competencies of health workers to provide integrated PMH care while also strengthening broader dimensions of care systems, including referral linkages, strategies to motivate health workers, and context-adapted care protocols [11].

### Strengths and limitations of the study

#### Strengths

The study’s strengths include its comprehensive setting, which covered diverse primary healthcare facilities, allowing for a broader understanding of PMH service integration. Maximum variation sampling ensured the gathering of diverse perspectives from maternal health workers, facility managers, and sub-city program managers, which enriched the depth of the data. The sequencing of interviews (from health workers to facility managers and then to sub-city health department managers) helped to refine the probing questions based on the initial responses. Member checking with participants also enhanced credibility of the findings.

#### Limitations

The study has several limitations. Despite efforts to reduce bias, the first author’s role as a technical support provider within the same sub-city could have influenced participants’ responses, potentially leading to social desirability bias or reluctance to criticize the system. Although interviews were conducted in private rooms at participants’ workplaces, we acknowledge that the setting may have influenced their responses, despite assurances of confidentiality and voluntary participation. Member checking was only performed for a portion of the transcripts. The study also had a limited number of participants from the private sector, reducing transferability of some findings to private facility contexts within similar settings. Additionally, while this study focused on the capacity and experiences of frontline workers and facility and sub-city level managers, it did not fully explore broader meso- and macro-level drivers, as perspectives of regional or national-level health system actors were not included. Future studies should explore these higher-level perspectives and private-sector contexts to have a comprehensive understanding of system-wide challenges and identify action points to improve the integration of PMH into routine MCH services.

## Conclusions

This study highlights the significant challenges that hinder maternal health workers from providing integrated PMH care, which are exacerbated by systemic, individual, and socio-cultural barriers. While maternal health workers recognize the importance of PMH, they often lack adequate knowledge, standardized protocols and resources. High workloads, limited training and weak referral systems further hindered the provision of integrated care. Cultural stigma and socio-cultural perceptions also discourage help-seeking, even among providers. Nevertheless, the findings reveal opportunities for improvement, such as in-service training, standardized tools, and better referral networks and reducing stigma. To advance the provision of integrated PMH care provision, a multi-pronged approach involving enhanced training, institutional support, community engagement and a strong commitment to policy to fully integrate PMH into routine maternal services is required.

## Supplementary Information

Below is the link to the electronic supplementary material.

Supplementary Material 1

## Data Availability

The dataset used for this study is available from the principal investigator (MB) upon reasonable request.
