# Supplementary material for: Maternal health workers’ knowledge, practices, and influencing factors in screening and managing perinatal mental health conditions in primary healthcare facilities in Addis Ababa, Ethiopia: a qualitative study
Source: BMC Health Serv Res. 2026 Mar 5;26:387. doi: 10.1186/s12913-026-14284-w (PMC13011635; doi:10.1186/s12913-026-14284-w)
Supplement: Supplementary file 1 — Supplementary Material 1 [file 12913_2026_14284_MOESM1_ESM.docx]

**Capacity of maternal health workers to provide integrated perinatal mental health services in Addis Ababa, Ethiopia: a qualitative study**

**In-depth interview Guide**

1. **Would you please tell me about mental health conditions women may experience during pregnancy, childbirth, and the postpartum period based on your general knowledge?**

Probe: Postpartum "blues," Postpartum depression, Anti-partum depression

1. **What are your sources of knowledge of these perinatal mental health conditions?**

Probe: Pre-service training, In-service training, Media sources, Colleagues, etc.

1. **What do you think are the contributors to poor mental health among women during the pregnancy and the postpartum periods?**

Probe: Identifying triggers and risk factors for mental health issues.

1. **What do you do for mothers who experience prenatal mental health conditions in your facility?**
2. **What do you think is the assessment and evaluation of women mental health statues of pregnant and lactating women?**

Probe: the approaches to assess mental health during this period

1. **What methods or tools do you or your colleagues use to assess the mental health status of pregnant and postpartum women?**

probe – checklists, and screening tools

**How often do you and your colleagues use these perinatal mental health screening tools?**

Probe – do they use the tool routinely for all women?

**How do you perceive your own competence and confidence in addressing prenatal mental health conditions?**

Probe – is there a gap in training, gaps in school curriculum

**What challenges do you experience in providing care (promotive, preventive, and treatment) for women with perinatal mental health for women with perinatal mental health?**

Probe: Community perceptions of mental health conditions, supportive environment at health facility level, religious and traditional healers, healthcare provider challenges.

**From your perspective, what actions are required to respond to the maternal mental health needs of pregnant and postpartum women?**

Probe: social support, counseling, health workers capacity building, management support, etc

**Do you have any additional insights, experiences, or thoughts on perinatal mental health problems?**
